# Physical fitness and incident mild cognitive impairment: a systematic review

**DOI:** 10.1186/s11556-025-00376-9

**Published:** 2025-06-14

**Authors:** Matteo Bergmann, Yonas Endale Geda, Klaus Boes, Alexander Woll, Janina Krell-Roesch

**Affiliations:** 1 Karlsruhe Institute of Technology, Karlsruhe, Germany; 2 Barrow Neurological Institute, Phoenix, USA

**Keywords:** Motor performance, Endurance, Cardiovascular fitness, Strength, Balance, Gait, Mobility, Mild cognitive impairment, Longitudinal

## Abstract

**Background:**

Higher physical fitness is associated with various health outcomes, including decreased dementia risk. Little is known as to whether physical fitness is also associated with new onset of mild cognitive impairment (MCI). Our aim was to provide an overview of longitudinal research on the associations between physical fitness and the risk of incident MCI.

**Methods:**

We conducted a systematic literature review that examined associations between different components of physical fitness such as strength or endurance with incident MCI in older adults. We searched PubMed, Scopus, and Web of Science databases for longitudinal and/ or prospective cohort studies published in English or German. Screening was performed independently by two authors, and quality of included studies was assessed using the Newcastle Ottawa Scale.

**Results:**

The search yielded 12,298 studies, of which 19 were included in the review, with follow-up times ranging from 2 to 26 years, and sample sizes ranging from 87 to 995,243 persons. Thirteen studies that examined associations between strength, variables related to muscle quality and function with incident MCI revealed inconsistent findings, e.g., six studies showed that lower handgrip strength was associated with higher MCI risk or that higher handgrip strength was associated with decreased MCI risk, respectively; while five studies reported no associations between handgrip strength and MCI risk or only for females. One study reported associations between lower cardiovascular fitness and increased risk of MCI. Twelve studies examined associations between balance, mobility and gait-related variables, mainly focusing on gait speed, but results were inconsistent, e.g., while some reported associations between slower gait speed and increased MCI risk, others did not or only in subgroups. Five studies reported associations between higher global/ composite fitness scores and decreased risk of incident MCI. Quality of included studies was rated as good.

**Conclusion:**

Higher cardiovascular and overall physical fitness is associated with a decreased risk of incident MCI. There are inconsistent associations between strength, balance- or gait-related variables and MCI risk. These findings indicate the importance of overall and cardiovascular physical fitness to potentially delay new onset of MCI. More research is needed to confirm these observations, and to untangle mechanisms underlying the associations between physical fitness components and MCI risk.

**Supplementary Information:**

The online version contains supplementary material available at 10.1186/s11556-025-00376-9.

## Introduction

Physical fitness is critical for both physical and mental health in old age. According to the American College of Sports Medicine (ACSM), one can distinguish between health-related (i.e., cardiorespiratory endurance, body composition, muscular strength, muscular endurance, and flexibility), and skill-related physical fitness components (i.e., agility, coordination, balance, power, reaction time, and speed) [1]. However, there is no universal definition of physical fitness. Other authors have proposed five different motor abilities as main components of physical fitness in humans, i.e., cardiorespiratory fitness or endurance, muscular strength, gross motor coordination, flexibility, and speed [2], or defined physical fitness as attributes enabling a person to perform activities that require aerobic capacity, strength, flexibility, or endurance [3]. It is well established that, in general, physical fitness components decline with increasing age (e.g., [4]), and older as compared to younger adults have reduced movement coordination, speed and lower-limb muscle strength, or impaired postural stability and balance, amongst others [5]. However, it has been proposed that certain fitness components may decline faster than others [6, 7], and that the decline in physical fitness varies considerably from person to person depending, for example, on the level of engaging in physical activity; leading to the construct of “fitness gap” [8]. Decreased physical fitness levels in older adults are associated with various health outcomes, including but not limited to increased mortality (e.g., [9]), metabolic syndrome (e.g., [10]), decreased activities of daily living and higher mobility-related disability (e.g., [11]), and higher risk of falling (e.g., [12]), particularly in persons with dementia [13] or mild cognitive impairment (MCI; [14, 15]).

MCI is the intermediate stage between normal cognitive aging and dementia, and persons with MCI are at increased risk of progression to dementia. MCI is characterized by cognitive concern expressed by a person, an informant and/ or a physician; impairment in one or more cognitive domains (i.e., memory, attention/ executive function, language, and visuospatial skills); essentially normal functional activities; and absence of dementia [16–18]. A recent meta-analysis estimated the global prevalence of MCI to be about 16% in community-dwelling persons aged ≥ 50 years [19]. Risk factors of MCI reported in the literature include advanced age, lower education, APOEε4 genotype, diabetes, neuropsychiatric symptoms, or cardiovascular disease [20]. In addition, we and others have shown that engaging in physical activity is associated with a decreased risk of new onset of MCI [21–25], albeit conflicting reports also exist [26].

A growing body of research examines the associations between different components of physical fitness and cognitive impairment in old age. With regard to associations between physical fitness and cognitive function in older adults, a large study among 877 persons aged ≥ 65 years showed that higher cardiorespiratory fitness is associated with better global and domain-specific cognitive performance [27], and a systematic review of longitudinal studies published before 2011 showed that physical functioning was associated with cognitive changes over time in older adults, with associations varying depending on the measurements used to assess both physical and cognitive performance [28]. Similarly, scoping reviews concluded that handgrip strength [29], slow gait speed [30], or walking ability [31] (mainly indicated by gait speed but also other parameters such as step frequency or variability) are associated with longitudinal cognitive decline, including dementia. Rather less reviews are available that focused on categorical outcomes of cognitive status, e.g., cognitive impairment or dementia. For example, a recent scoping review noted that impaired upper limb motor function, i.e., slower speed, increased errors, and greater movement variability, is associated with cognitive impairment, but most participants of included studies were recruited from clinical settings [32]. In addition, a systematic review and meta-analysis, mainly including prospective cohort studies, showed that decreased lower limb motor function as well as reduced performance on composite motor function, balance and gait velocity are associated with an increased risk of incident dementia [33]. Furthermore, a descriptive review revealed associations between different physical functions such as gait, balance, or fine and gross motor skills, with declines in cognitive function and risk of new onset of cognitive impairment [34]. However, most reviews have been published more than 10 years ago, and to the best of our knowledge, no review in recent years has examined the associations between various components of physical fitness as predictors, and the risk of incident MCI as outcome of interest in older adults.

In this review, we sought to provide an overview of the current state of observational research, i.e., longitudinal and prospective cohort studies on the associations between physical fitness or motor performance, and the risk of new onset of MCI. We anticipate that this review may generate important information for researchers, clinical practitioners, patients, and care partners alike on the importance of maintaining fitness or engaging in fitness-enhancing activities in old age to potentially delay new onset of MCI.

## Methods

### Design

This systematic review was registered in the International Prospective Register of Systematic Reviews (PROSPERO; ID: 467990), and was carried out based on the Preferred Reporting Items for Systematic Reviews and Meta-Analyses (PRISMA) expanded checklist [35].

### Search strategy

We searched PubMed database using a predefined search term that included terms related to motor performance or physical fitness and MCI. Since relying solely on database search strategies may be non-exhaustive [36], further relevant studies were identified by screening the reference lists of all selected articles. The literature search was conducted in August of 2023, updated in September of 2024 and March of 2025, at which time we also searched Scopus and Web of Science databases in addition to PubMed. For the full search terms by database, please refer to Supplementary material 1.

### Inclusion and exclusion criteria

Inclusion and exclusion criteria were formulated according to PECO as follows: (1) Population: We included studies among older persons free of cognitive impairment at baseline. Studies in specific patient groups (e.g., post-stroke patients) were excluded. (2) Exposure: We included studies that assessed or considered parameters of physical fitness (e.g., strength, endurance, gait etc.) as predictors of interest (i.e., independent variables). We did not include studies that examined parameters related to body composition/ weight, albeit body composition is regarded as health-related fitness parameter by some authors. (3) Comparator: Not applicable. (4) Outcome: We included studies that assessed or considered incident/ new onset of MCI as outcome of interest (i.e., dependent variable).

With regard to study design, we included all observational studies with longitudinal design (e.g., prospective cohort study) regardless of follow-up time or period, and published in English or German. Intervention studies, case reports or study protocols, as well as (systematic) reviews were excluded.

### Study selection

For the initial search, all identified studies were exported to www.rayyan.ai (AI-based reference management tool), and duplicates were removed. Screening was performed independently by two authors (MB & JKR). First, studies were included/ excluded based on their titles. Second, all included studies were transferred to Citavi®, and included/ excluded based on their abstracts. Third, the full texts of included studies were scrutinized, and any reasons for exclusion were documented by the two authors. Any disagreements at one of the screening stages were addressed via discussion until consensus was reached. For the updated search, all retrieved articles were uploaded to ASRreview (AI-based reference management tool), where title and abstract screening was conducted. The remaining articles were then imported to Zotero, where full-text screening were performed.

### Assessment of study quality

Quality of included studies in terms of reporting was assessed by two authors (MB & JKR) using the Newcastle-Ottawa Scale checklist. Studies were rated on a scale of 0 to 9 points, based on three categories (selection, comparability and outcome). A higher number of points reflects a better study quality.

### Data extraction

All relevant information/ data from included studies was extracted using Microsoft Excel and Word, and comprised details on study characteristics such as sample size, study participants/ population and cognitive status, follow-up duration, predictor variables (i.e., physical fitness), outcome variable (i.e., incident/ new onset of MCI), and results as well as any strengths and limitations of the studies.

## Results

**Fig. 1:**
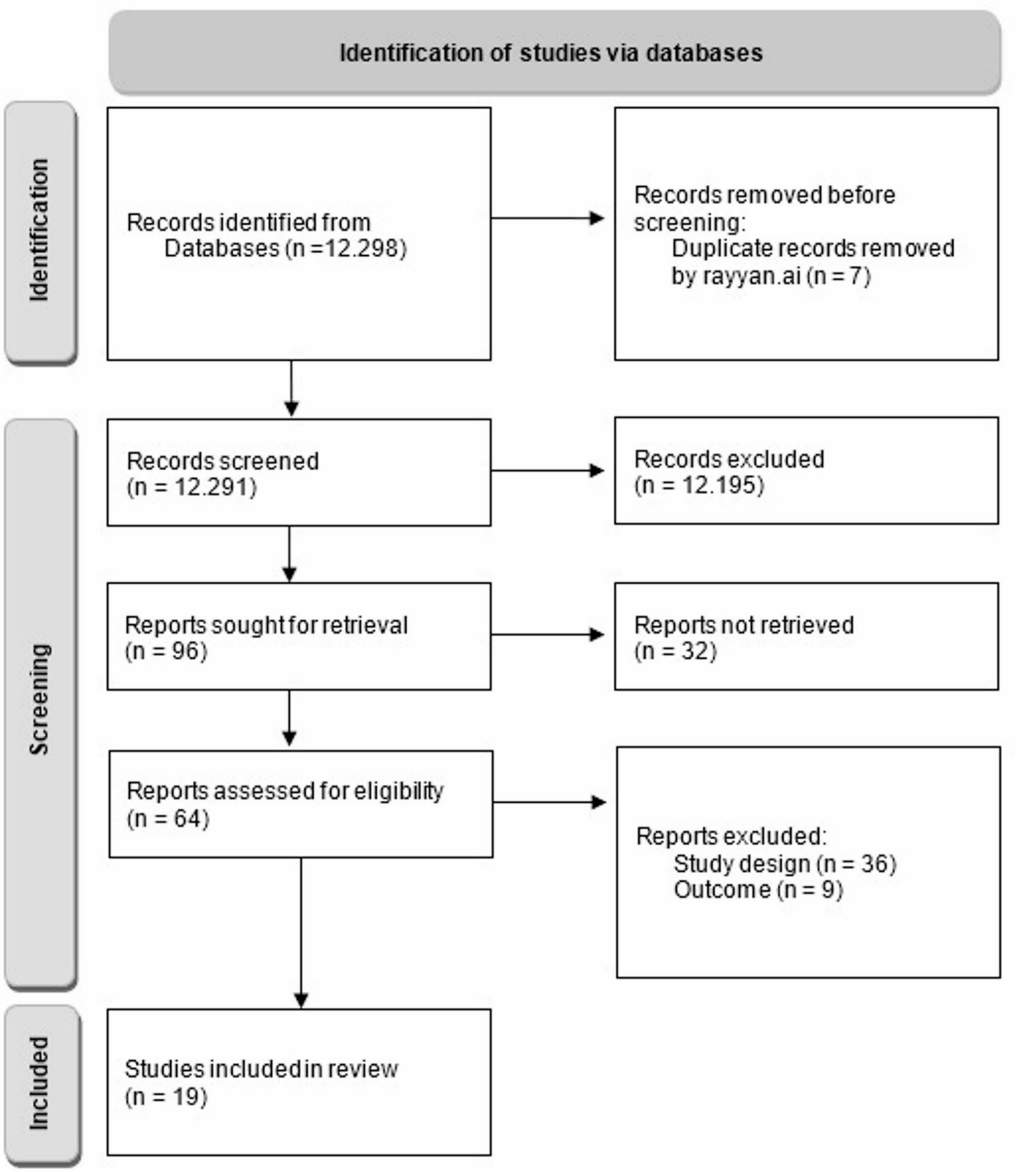
Flowchart of screening process

The search yielded 12,298 studies, of which 19 were finally included in the review (Fig. 1 provides a flowchart of study selection). 17 out of 19 studies were conducted in a population-based setting, and follow-up times ranged from 2 [37, 38] to 26 [39] years. Studies were conducted in four different continents: North-America (i.e., USA and Mexico), Europe (i.e., the Netherlands, Sweden, Germany), Australia, and Asia (i.e., China, South Korea and Singapore). Sample sizes ranged between 87 [40] and 995,243 [39] persons, with a total of 1,036,186 persons from all studies included in this review. Measures of physical fitness used in studies and for which associations with MCI were reported were: (1) strength and variables related to muscle quality and function (i.e., handgrip strength [37, 38, 41–50], skeletal muscle mass [41, 44, 46], hand dexterity [42], leg strength [51], composite strength score [52]); (2) endurance/ cardiovascular fitness (i.e., graded cycle ergometer test [39]); (3) balance (i.e., one leg stand [42, 47]), mobility (i.e., Timed Up and Go test [51], Performance Oriented Mobility Assessment (POMA) [51]) and gait-related variables (i.e., speed, function, variability [37, 40, 42, 43, 46, 49–51, 53–55]); and (4) global or composite physical fitness scores including at least two of categories 1–3 such as strength and balance or gait [42, 46, 49] or Short Physical Performance Battery (SPPB) [44, 45]. For an overview of all studies included in the review, please refer to Table 1. For a cross table providing an overview of variables examined in included studies, please refer to Supplementary material 2.

**Table 1:** Overview of included studies

Author (year), country	Study setting	Study sample	Fitness (predictor variables)	Incident MCI (outcome variable)	Main results
Beeri et al. (2021a), USA [41]	Rush Memory and Aging Project (longitudinal cohort study) Mean follow-up: 4.9 years (for analyses on incident MCI)	Overall study sample: *N* = 1175 (Mean age = 80.9 years; 77% Females; 95% White) Subsample for incident MCI: *N* = 816 Participants: Chicago metropolitan area; residents of senior house facilities, retirement communities/ homes, etc.	Sarcopenia: derived from skeletal muscle mass and muscle function Muscle mass index: derived from bioelectrical impedance analysis Muscle function: grip strength from hand dynamometer; gait speed (m/s)	Annual uniform clinical evaluation including medical history, neurologic examination, MMSE and neuropsychological testing (17 tests → global cognitive score and five domain-specific scores) Diagnosis of MCI required cognitive impairment in absence of dementia. MCI classified as amnestic and non-amnestic	316 participants developed incident MCI (38.7%) Cox proportional hazards model, controlling for age, sex, education, race, and height squared • Composite sarcopenia: HR 1.21 (95% CI 1.01–1.45), *p* = 0.04 • Grip strength: HR 0.88 (95% CI 0.75–1.02), *p* = 0.09 • Muscle mass: HR 0.91 (95% CI 0.80–1.04), *p* = 0.16 • Composite sarcopenia + grip strength: HR 0.89 (95% CI 0.76–1.03), *p* = 0.13 • Composite sarcopenia + grip strength + muscle mass: HR 0.92 (95% CI 0.81–1.05), *p* = 0.24 Association of sarcopenia with incident MCI - was not attenuated by number of vascular risk factors and vascular diseases; - did not vary with age, sex, education, race, vascular risk factors, or diseases
Beeri et al. (2021b), USA [42]	Religious Orders Study; Rush Memory and Aging Project; Minority Aging Research Study (longitudinal cohort studies) Mean follow-up: 7.3 years (for analyses on incident MCI)	Overall study sample: *N* = 1160 (Mean age = 73.2 years; 78% Females; 50% African Americans) Subsample for incident MCI: *N* = 913 Participants: drawn from 3 ongoing studies	Global motor score and four specific motor abilities scores 1) Hand Strength (bilateral grip strength; pinch strength) 2) Hand Dexterity (number of pegs placed in the Purdue Pegboard in 30 s; finger tapping in 10 s) 3) Gait Function (time and number of steps to walk eight feet and turn 360°) 4) Leg Strength and Balance (10 s one leg stand; 10 s toes stand)	Annual uniform clinical evaluation including medical history, neurologic examination, MMSE and neuropsychological testing (17 tests → global cognitive score and five domain-specific scores) Diagnosis of MCI required cognitive impairment in the absence of dementia. MCI classified as amnestic and non-amnestic	335 participants developed incident MCI (36.7%) Cox proportional hazards model, controlling for age, sex, education, and race (individual association between motor function and incident MCI) • Global motor score: HR 0.79 (95% CI 0.68–0.92), *p* = 0.002 • Hand dexterity: HR 0.86 (95% CI 0.80–0.93), *p* < 0.001 • Hand strength: HR 0.93 (95% CI 0.89–0.97), *p* < 0.001 • Gait function: HR 0.91 (95% CI 0.87–0.96), *p* < 0.001 • Leg strength: HR 1.00 (95% CI 0.99-1.00), *p* = 0.295 Associations of global motor score with incident MCI did not vary by age, sex, education, or race; adjustments for cardiovascular risk factors/ diseases, APOEε4 genotype or baseline MMSE did not alter the association Associations between global motor function and MCI subtypes: Incident non-amnestic MCI: HR 0.91 (95% CI 0.82–0.99), *p* = 0.04; incident amnestic MCI: HR 0.92 (95% CI 0.83–1.01), *p* = 0.08 Cox proportional hazards model, controlling for age, sex, education, and race (association of different combinations of motor function and incident MCI) • Hand strength (HR 0.94 (95% CI 0.90–0.98), *p* = 0.005) + gait function (HR 0.91 (95% CI 0.87–0.96), *p* = 0.001) • Hand strength (HR 0.95 (95% CI 0.91–0.99), *p* = 0.023) + gait function (HR 0.93 (95% CI 0.88–0.98), *p* = 0.008) + hand dexterity (HR 0.92 (95% CI 0.83–1.01), *p* = 0.071) • Hand strength (HR 0.95 (95% CI 0.91–0.99), *p* = 0.015) + gait function (HR 0.93 (95% CI 0.87–0.98), *p* = 0.011) + hand dexterity (HR 0.90 (95% CI 0.81–0.99), *p* = 0.035) + leg strength (HR 1.00 (95% CI 1.00-1.01), *p* = 0.304)
Boyle et al. (2009), USA [52]	Rush Memory and Aging Project (longitudinal cohort study) Mean follow-up: 3.6 years (for analyses on incident MCI)	Overall study sample: *N* = 970 (Mean age 80.3 years; 33% Females; 92% Whites) Subsample for incident MCI: *N* = 694 Participants: Chicago metropolitan area; residents of senior house facilities, retirement communities/ homes, etc.	Muscle strength: Composite measure derived from tests in 11 muscle groups (upper extremities - abduction, flexion, extension in both arms; lower extremities - hip flexion, knee extension, plantar flexion, ankle dorsiflexion in both legs; bilateral grip and pinch strength; axial strength (maximum inspiratory and expiratory pressure)	Annual uniform clinical evaluation including medical history, neurologic examination, and neuropsychological testing (21 tests including MMSE) Diagnosis of MCI required cognitive impairment in the absence of dementia	275 participants developed MCI (39.6%) Cox proportional hazards model adjusted for age, sex, and education • Muscle strength: incident MCI - HR 0.67 (95% CI 0.54–0.84), *p* < 0.05; persistent MCI (i.e., MCI, dementia, or death at a subsequent evaluation) - HR 0.55 (95% CI 0.38–0.79), *p* < 0.05
Boyle et al. (2010), USA [49]	Rush Memory and Aging Project (longitudinal cohort study) Mean follow-up: 6 years	Overall study sample: *N* = 761 (Mean age 79 years; 76% Females; 89% Whites and non-Hispanic) Participants: Chicago metropolitan area; residents of senior house facilities, retirement communities/ homes, etc.	Physical frailty: Composite measure based on four components 1) Grip strength assessed using hand dynamometer 2) Gait speed based on time to walk eight feet 3) Body composition through BMI 4) Fatigue based on two questions of the CES-D Scale	Annual uniform clinical evaluation including medical history, neurologic examination, and neuropsychological testing (21 tests including MMSE) Diagnosis of MCI required cognitive impairment in the absence of dementia assessed by a neuropsychologist	305 participants developed MCI (40%) Cox proportional hazards model adjusted for age, sex and education • Physical frailty: incident MCI - HR 1.63 (95% CI 1.27–2.08), *p* < 0.05; persistent MCI (MCI present on consecutive examinations) - HR 1.65 (95% CI 1.15–2.36), *p* < 0.05 • Physical frailty: incident MCI (controlled for all covariates simultaneously) - HR 1.59 (95% CI 1.21–2.10), *p* < 0.05; persistent MCI (controlled for all covariates simultaneously) - HR 1.63 (95% CI 1.08–2.44), *p* < 0.05 Cox proportional hazards model on individual frailty components adjusted for age, sex and education • Grip strength: incident MCI - HR 1.28 (95% CI 1.07–1.54), *p* < 0.05; persistent MCI: HR 1.34 (95% CI 1.02–1.75), *p* < 0.05 • Gait speed: incident MCI - HR 1.27 (95% CI 1.11–1.45), *p* < 0.05 • BMI: incident MCI - HR 1.01 (95% CI 0.89–1.16), *p* > 0.05 • Fatigue: incident MCI - HR 1.10 (95% CI 0.98–1.24), *p* > 0.05
Byun et al. (2018), South Korea [40]	Korean Longitudinal Study on Cognitive Aging and Dementia (prospective cohort study) Mean follow-up: 3.9 years	Overall study sample: *N* = 91 (Mean age 67 years; 44% Females) Subsample for incident MCI: *N* = 87 Participants: Community-dwelling cognitively normal older individuals aged ≥ 60 years without dementia, cerebral ischemic burden or Parkinsonism	Gait assessments: Cadence, step time, step length, speed, and gait variability assessed using tri-axial accelerometer during walking at self-selected pace on a flat straight walkway	Uniform clinical evaluation, including medical history, physical and neurological examinations, and laboratory tests (e.g., APOE genotyping) every 2 years Diagnosis of MCI made by consensus panel based on acquired data from neuropsychological testing and based on International Working Group on MCI criteria	7 participants developed MCI (8%) Kaplan-Meier and Cox proportional hazard regression analyses adjusted for age, sex, education, CIRS score, GDS score, and APOEε4 • Gait speed only (mid-high tertiles = reference) - Low tertile: HR 2.95 (95% CI 0.44–19.50), *p* = 0.263 • Gait variability only (mid-high tertiles = reference) - Low tertile: HR 10.35 (95% CI 1.16–92.40), *p* = 0.036 • Gait speed + variability (mid-high tertiles = reference) - Speed low tertile: HR 5.04 (95% CI 0.53–48.18), *p* = 0.161; Variability low tertile: HR 11.97 (95% CI 1.29-111.37), *p* = 0.029 Differences in MCI risk by gait variability group (χ2 = 9.64, *p* = 0.002, log-rank test): Mean MCI-free survival in high variability group 12% shorter than in mid-to-low tertile group Comparable MCI risk between gait speed groups (51.59 ± 0.70 vs. 50.64 ± 1.77 months; χ2 = 1.16, *p* = 0.281) Supplementary analyses revealed no sex effect; confirmed associations between gait variability status and MCI risk when additionally adjusting for baseline MMSE score (high variability: HR = 21.28, 95% CI = 1.18–384.14); showed that risk of incident MCI increased 1.5 times per 10% increment of gait variability, whereas it did not change with changes of gait speed
Feng et al. (2023), China [38]	Longitudinal study Mean follow-up: 2 years	Overall study sample: *N* = 2663 (Mean age 56.6 years; 65% Females) Subsample for incident MCI: *N* = 743 Participants: Community-dwelling adults aged ≥ 35 years from rural areas in Fuxin, Liaoning Province, China	Grip strength assessed using hand dynamometer, mean grip strength calculated from three attempts per hand.	Evaluation using Chinese version of Montreal Cognitive Assessment-Basic (MoCA-BC) every 2 years Diagnosis of MCI based on criteria proposed by NIA-AA Working Group	127 participants developed MCI (17%) Binary logistic regression model adjusted for age, baseline MOCA-BC score, ethnicity, education, income, BMI, smoking, drinking, physical labor level, hypertension, diabetes, dyslipidemia and coronary heart disease • Handgrip strength (males; per 5 kg decreased): OR 0.90 (95% CI 0.64–1.26), *p* = 0.53 • Handgrip strength (females; per 5 kg decreased): OR 1.45 (95% CI 1.11–1.92), *p* = 0.007 Association between quintile of handgrip strength and MCI stratified by age group (35–60 years): Males: • Quintile 4: OR 1.22 (95% CI 0.67–2.22), *p* = 0.51 • Quintile 3: OR 1.62 (95% CI 0.87-3.00), *p* = 0.126 • Quintile 2: OR 2.02 (95% CI 1.01–4.04), *p* = 0.046 • Quintile 1: OR 4.86 (95% CI 1.81–13.73), *p* = 0.002 Females: • Quintile 4: OR 1.26 (95% CI 0.85–1.88), *p* = 0.25 • Quintile 3: OR 1.10 (95% CI 0.72–1.67), *p* = 0.66 • Quintile 2: OR 0.88 (95% CI 0.56–1.39), *p* = 0.6 • Quintile 1: OR 1.79 (95% CI 1.08–2.99), *p* = 0.025 Association between quintile of handgrip strength and MCI stratified by age group (> 60 years): Males: • Quintile 4: OR 1.60 (95% CI 0.60–4.33), *p* = 0.35 • Quintile 3: OR 1.92 (95% CI 0.76–4.94), *p* = 0.17 • Quintile 2: OR 1.04 (95% CI 0.42–2.59), *p* = 0.93 • Quintile 1: OR 2.36 (95% CI 0.95-6.00), *p* = 0.07 Females: • Quintile 4: OR 1.46 (95% CI 0.63–3.44), *p* = 0.38 • Quintile 3: OR 1.08 (95% CI 0.48–2.46), *p* = 0.86 • Quintile 2: OR 0.88 (95% CI 0.40–1.99), *p* = 0.76 • Quintile 1: OR 1.73 (95% CI 0.80–3.84), *p* = 0.17 Supplementary analyses revealed that the interaction term between sex and handgrip strength on incident MCI was significant (*p* = 0.015)
Hooghiemstra et al. (2017), The Netherlands [37]	Clinical Course of Cognition and Comorbidity Study (longitudinal multicenter study) Mean follow-up: 2.1 years (for analyses on incident MCI)	Overall study sample: *N* = 309 (Mean age 69.6 years; 35% Females) Subsample for incident MCI: *N* = 141 (with subjective cognitive impairment at baseline) Participants: recruited from three different university memory clinics from Amsterdam and Maastricht, aged ≥ 55 years and free of dementia	Gait speed: Fast pace over a distance of 15 feet in sec; performed indoors on flat surface; participants were allowed to use assistive devices when needed; distance was walked twice and mean gait speed (m/s) was calculated Grip strength: hydraulic hand dynamometer; mean grip strength calculated from two attempts while standing or sitting	Annual uniform clinical evaluation including medical, neurological, and neuropsychological examination. Diagnosis of MCI based on clinical judgment, following Petersen criteria. Diagnoses were re-evaluated by a multidisciplinary team	23 participants developed MCI (16%) Cox proportional hazard models adjusted for Model 1: age, gender, education, and center; Model 2 additionally adjusted for comorbidities (CIRS-G cardiac and vascular sub scores) • Gait speed (Model 2): HR 1.01 (95% CI 0.65–1.56), p = n.s. • Grip strength (Model 2): HR 1.13 (95% CI 0.63–2.03), p = n.s. Slower gait speed associated with increased risk of incident MCI in persons aged > 65 years (Model 2: HR 1.84, 95% CI 1.14–2.99), but not in those aged ≤ 65 years (Model 2: HR 0.85, 95% CI 0.48–1.52)
Lipnicki et al. (2017), Australia [55]	Sydney Memory and Ageing Study (longitudinal cohort study) Mean follow-up: 6 years	Overall study sample: *N* = 873 (Mean age 78.7 years; 56% Females) Subsample for incident MCI: *N* = 504) Participants: Community-dwelling older adults aged between 70 and 90 years and free of dementia	Gait speed: assessed using 6-meter walk test	Neuropsychological test battery administered by trained psychologist every 2 years Diagnosis of MCI required: self or informant complaint of memory or other cognitive function decline; objective cognitive impairment (at least one test score ≥ 1.5 SD below published normative values, adjusted for age and/ or education where possible); no dementia; no or minimal impairment in instrumental ADL attributable to cognitive impairment	94 participants developed MCI (18.7%) Multinomial logistic regressions adjusted for age, sex, and education • Gait speed: OR 1.14 (95% CI 1.05–1.24), *p* < 0.001
Luo et al. (2022), USA [43]	Study of Osteoporotic Fractures (prospective cohort study) Follow-up: 20 years	Overall study sample: *N* = 9268 (Mean age 71.7 years; 100% Females; 96% White) Subsample for incident MCI: *N* = 8304 Participants: Community-dwelling women aged ≥ 65 years from Baltimore, Minneapolis, Pittsburgh and Portland	Handgrip strength in kilograms: using isometric dynamometer, average of both hands Gait speed in m/s: 6-meter walk at usual pace	Modified MMSE administered at baseline and 5 follow-up visits over 20 years Diagnosis of MCI required a m-MMSE score of 19–22. A score of 23–26 was considered to reflect normal cognition	No information provided as to how many participants developed MCI Multi-state models adjusted for age, education, BMI, physical activity, alcohol consumption, smoking, history of hypertension, diabetes, and cardiovascular disease Progression from cognitively normal to MCI • Gait speed: HR 0.50 (95% CI 0.37–0.67), *p* < 0.05 • Handgrip: HR 0.96 (95% CI 0.95–0.97), *p* < 0.05 Progression from cognitively normal to death • Gait speed: HR 0.21 (95% CI 0.15–0.29), *p* < 0.05 • Handgrip: HR 0.99 (95% CI 0.98–1.01), *p* < 0.05
Moon et al. (2016), South Korea [44]	Korean Longitudinal Study on Health and Aging (prospective cohort study) Mean follow-up: 5 years	Overall study sample *N* = 297 (Mean age 71.9 years; 47% Female) Participants: Community-dwelling older adults from Korea aged ≥ 65 years and free of cognitive impairment at baseline	Sarcopenia: Composite measure based on muscle mass, muscle strength, and physical performance 1) Muscle mass: Appendicular lean mass in both arms and legs using dual-energy x-ray absorptiometry 2) Muscle strength: Handgrip strength measured with a handgrip dynamometer; average value from two attempts 3) Physical performance: SPPB to assess lower extremity performance (standing balance, chair stand, and gait speed)	Cognitive evaluation using Korean versions of Consortium to Establish a Registry for Alzheimer’s Disease Clinical Assessment Battery and Mini International Neuropsychiatric Interview Diagnosis of MCI made by consensus panel based on acquired data from neuropsychological testing and based on International Working Group on MCI criteria	50 participants developed MCI (16.8%), 5 developed dementia and were also included in the analyses Multivariate binary logistic regression analysis adjusted for sex, age, education, MMSE, GDS, CIRS • Muscle mass: HR 0.82 (95% CI 0.38–1.75), *p* = 0.61 • Muscle strength: HR 1.34 (95% CI 0.28–6.37), *p* = 0.72 • Physical performance: HR 2.22 (95% CI 1.05–4.72), *p* = 0.04
Ng et al. (2022), Singapore [51]	Singapore Longitudinal Ageing Study (prospective cohort study) Mean follow-up: 4.5 years	Overall study sample *N* = 2544 (Mean age 65.4 years; 65% Female) Subsample for incident MCI: 1208 Participants: Community-dwelling older adults from South West and South-Central regions of Singapore aged ≥ 55 years	Physical and functional performance: Composite measure based on 4 different components: 1) TUG: Fastest time from two attempts 2) Gait speed: Time in sec taken to walk 6 m at fastest pace 3) KES: maximum isometric contraction of the dominant leg 4) POMA to measure dynamic and static balance	Clinical evaluation including Mini Mental Status Exam (MMSE), Clinical Dementia Rating (CDR) scale, and neurocognitive evaluation using standardized tests for memory, executive function, language, visuospatial skills and attention. Diagnosis of MCI made by consensus panel based on acquired data from neuropsychological testing and based on International Working Group on MCI criteria	60 participants developed MCI (5%), 6 developed dementia and were also included in the analyses Logistic regression model adjusted for Model 1: age, sex, education, smoking, physical activity, social activity, productive activity, multi-morbidity, metabolic syndrome; Model 2: additionally adjusted for MMSE Model 1: • TUG (per SD increase): OR 1.60 (95% CI 1.07–2.38), *p* < 0.05 • Gait speed (rev.; per SD increase): OR 1.51 (95% CI 1.07–2.11), *p* < 0.05 • POMA (rev.; per SD increase): OR 1.39 (95% CI 0.85–2.26), p = n.s. • KES (rev.; per SD increase): OR 1.14 (95% CI 0.81–1.62), p = n.s. Model 2: • TUG (per SD increase): OR 1.52 (95% CI 1.01–2.31), *p* < 0.05 • Gait speed (rev.; per SD increase): OR 1.53 (95% CI 1.08–2.16), *p* < 0.05 • POMA (rev.; per SD increase): OR 1.34 (95% CI 0.80–2.23), p = n.s. • KES (rev.; per SD increase): OR 1.09 (95% CI 0.76–1.56), p = n.s. Significant associations between POMA and KES with incident MCI only in unadjusted models
Nyberg et al. (2014), Sweden [39]	Study of 18-year-old Swedish males, who enlisted for mandatory military service from 1968–2005 (i.e. born between 1950 and 1987 Mean follow-up: 25.7 years	Overall study sample *N* = 1,353,723 (Mean age 18 years; 0% Females) Subsample of *N* = 1,174,483 for analyses using cardiovascular fitness as predictor variables (*N* = 995243 used for final analyses) Participants: Swedish 18 years old males who enlisted for mandatory military service	Cardiovascular fitness: Graded cycle ergometer test assessing maximal work rate divided by bodyweight. W_max_/kg was then transformed to a score of 1 to 9, with 1 indicating lowest and 9 indicating maximal performance	Standardized psychological examination by physicians and psychologists during conscription examination. Diagnosis of MCI based on criteria according to the ICD	213 participants developed MCI (0.02%) Cox proportional hazards model adjusted for age, calendar year, BMI, region, conscription test center, parental education, own education, cognitive performance at age 18 • Low cardiovascular fitness: HR ranging between 2.96 (95% CI 1.83–4.78; adjusted for calendar year, BMI, region, conscription test center, parental education, cognitive performance at age 18) and 3.83 (95% CI 2.39–6.12; adjusted for calendar year, BMI, region, conscription test center) depending on type of adjustment • Medium cardiovascular fitness: HR ranging between 1.52 (95% CI 1.11–2.07; adjusted for calendar year, BMI, region, conscription test center, parental education, cognitive performance at age 18) and 1.75 (95% CI 1.28–2.38; adjusted for calendar year, BMI, region, conscription test center) depending on type of adjustment - Low and medium cardiovascular fitness associated with increased risk of incident MCI in subgroup analyses, i.e., among persons with cerebrovascular disease, diabetes or hypertension prior to MCI - Low performance in both cognitive and cardiovascular fitness tests associated with 48-fold increased risk of MCI (high performance in cognitive and cardiovascular fitness tests = reference group)
Pellecchia et al. (2022), USA [45]	World Trade Center Aging Study (prospective cohort study) Follow-up: Baseline assessment May 2016–April 2017, follow-up at least once before Dec 2019	Overall study sample *N* = 2687 (Mean age 53 years; 8% Females; 79% White) Subsample for analysis on incident MCI: *N* = 2170 Participants: World Trade Center responders	Physical functional impairment assessed through two tests: Lower extremity physical function derived from SPPB consisting of three components (balance, gait speed, and repetitive chair stand) Upper extremity physical function derived from handgrip strength while sitting upright in a chair and measured for both hands	MCI diagnosis based on NIA-AA diagnostic guidelines. MoCA score of 21–23	No information provided as to how many participants developed MCI Cox proportional hazards model adjusted for sex, race/ ethnicity, educational attainment, occupation, early arrival, ergonomic exposures, hypertension, diabetes, pulmonary embolism, and cancer • Handgrip strength (SD): HR 1.35 (95% CI 1.10–1.66), *p* = 0.004 • Lower extremity physical functional impairment: HR 1.55 (95% CI 1.21–1.98) *p* < 0.001 Physical functional impairment plays an intermediary role in relationship between PTSD and MCI, i.e., relationship between PTSD and incident MCI no longer significant after adjusting for physical functional impairment
Rosso et al. (2017), USA [53]	Healthy Aging and Body Composition (longitudinal cohort study) Total follow-up: 14 years	Overall study sample *N* = 193 (Mean age 73 years; 58% Females; 65% White) Participants: Community-dwelling older adults living in Memphis, TN or Pittsburgh, PA	Gait speed: Repeated measures of time to walk 6 m at usual pace. The investigators calculated increases in sec (slopes) to walk per year, with higher values indicating faster slowing of gait speed over 14 years	Clinically adjudicated cognitive diagnosis based on data from neuropsychological evaluation, neurological evaluation	69 participants developed MCI (35.8%) Logistic regression analyses adjusted for gait intercept, age, sex, race, education, coronary heart disease, diabetes mellitus, hypertension, recurrent falls, knee pain, quadriceps strength, and white matter hyperintensities Gait slowing: OR 1.45 (95% CI 0.99–2.15), p = n.s. Associations between gait slowing and cognitive impairment stronger in APOEε4 allele carriers
Salinas-Rodriguez et al. (2021), Mexico [46]	WHO Study on Global AGEing and Adult Health (longitudinal cohort study) Total follow-up: 8 years across three assessment waves	Overall study sample *N* = 496 (Mean age 65.5 years; 65% Females) Participants: Individuals aged ≥ 50 years from Mexico	Sarcopenia: Composite measure based on three components Skeletal muscle mass index derived from appendicular skeletal muscle mass. Low skeletal muscle mass (LSMM) was established by lowest quintile of skeletal muscle index based on sex-stratified Slow gait speed: 4 m time walk; lowest quintile of walking speed Handgrip strength: Average value of two handgrip measurements of dominant hand; weak handgrip defined as < 30 kg for men and < 20 kg for women	Standardized cognitive testing for verbal learning and recall, attention and working memory and executive function at each of the three waves Diagnosis of MCI required: 1) Concern regarding change in cognition 2) Evidence of impairment in one or more cognitive domains 3) Independence in basic ADL evaluated with Katz scale 4) No diagnosis of dementia	No information provided as to how many participants developed MCI Logistic and linear mixed-effects regression models adjusted for follow-up time duration, baseline MCI or cognitive function and various covariates including sociodemographic factors, health factors, and lifestyle factors Presence of sarcopenia: OR 1.74 (95% CI 1.02–2.96), *p* = 0.04 Components of sarcopenia: • Slowness: OR 1.57 (95% CI, 0.91–2.70), *p* = 0.11 • Handgrip weakness: OR 1.03 (95% CI 0.60–1.75), *p* = 0.92 • LSMM: OR 1.11 (95% CI 0.52–2.37), *p* = 0.78 • Slowness + handgrip weakness: OR 1.43 (95% CI 0.64–3.21), *p* = 0.38 • Slowness + LSMM: OR 0.94 (95% CI 0.38–2.34), *p* = 0.90 • Handgrip weakness + LSMM: OR 1.50 (95% CI 0.64–3.51), *p* = 0.35
Sattler et al. (2011), Germany [47]	German Interdisciplinary Longitudinal Study on Adult Development and Aging (prospective cohort study) Mean follow-up: 12 years	Overall study sample *N* = 381 Mean age at follow-up 74 years; 50% Females Subsample for incident MCI: *N* = 300 Participants: German adults who were born between 1930 and 1932 and 1950–1952	Balance assessed through “one-foot balance test”; participants balanced on one foot for 15 s Grip strength using “Martin-Vigorimeter”; participants press a ball alternating between dominant and non-dominant hand for four trials	Clinical evaluation including medical interviews, physical, and neurological examination MCI diagnosed according to the aging-associated cognitive decline criteria, and made by a consensus conference consisting of two psychiatry specialists under supervision of a specialist in Old Age Psychiatry	102 participants developed MCI (34%) Logistic regression models adjusted for education, socioeconomic status, gender, and depressive symptoms • Passing balance test: OR 0.35 (95% CI = 0.19–0.66), *p* < 0.01 • Handgrip strength: OR 1.00 (95% CI 0.99–1.01), p = n.s.
Tian et al. (2021), USA [54]	Baltimore Longitudinal Study of Aging (prospective cohort study) Mean follow-up: 7.3 years	Overall study sample *N* = 520 (Mean age 73 years; 51% Females; 71% White) Participants: Cognitively unimpaired persons aged ≥ 60 years from Baltimore, MD	Gait speed: time to walk 6 m at usual pace, 2 trials, faster time used for analysis	Uniform clinical evaluation including comprehensive health, cognitive, neurological and functional examinations Diagnosis of MCI based on Petersen criteria	64 participants developed MCI or AD (12.3%) Cox proportional hazards regression models adjusted for baseline age, sex, race and ethnicity, educational level, body mass index, total daily activity, and APOEε4 carrier status • Each 0.05 m/s slower gait speed: HR 1.07 (95% CI 1.00-1.15) *p* = 0.04 Activity fragmentation (i.e., degree to which an individual alternates physical activity bouts and periods of rest) may interact with gait in predicting MCI risk • Gait speed and activity fragmentation (interaction): HR 0.92 (95% CI 0.87–0.98) *p* = 0.01 • Each 0.05 m/s slower gait speed at low activity fragmentation: HR 1.19 (95% CI 1.07–1.32) • Each 0.05 m/s slower gait speed at high activity fragmentation: HR 1.01 (95% CI 0.93–1.10)
Werneck et al. (2023), Brazil [48]	Survey of Health, Ageing, and Retirement in Europe (prospective cohort study) Mean follow-up: 10.2 years	Overall study sample *N* = 19,686 (Mean age 64.9 years; 58% Female) Participants: Community dwelling adults aged ≥ 50 years from 14 different European countries	Handgrip strength: Hydraulic hand dynamometer assessed for both hands; age and gender standardized mean of each hand	MCI assessed using two different cognitive tests, i.e., memory recall test (immediate and delayed recall) and animal fluency task; age and education standardized; mean z-score of three tests to reflect global cognition. Participants performing 1.5 SD below mean considered as having MCI	1134 participants developed MCI (5.8%) Cox regression model adjusted for gender, age, country, education, presence of chronic diseases, elevated depressive symptoms, limitations in activities of daily living, body mass index, and cognition at baseline Models including MVPA (more than once a week) and handgrip strength • Handgrip strength (10% increase): HR 0.94 (95% CI 0.92–0.97) • MVPA: HR 0.87 (95% CI 0.76-1.00) Handgrip strength partly mediated association of MVPA with MCI (coefficient: 0.03; 95%CI: 0.01–0.05)
Yang et al. (2025), China [50]	Shandong Rural Elderly Health Cohort (longitudinal cohort study) Mean follow-up: 3 years	Overall study sample *N* = 3110 (Mean age 70.1 years; 64% Female) Participants: Community-dwelling adults aged 60 years and older from rural Shandong Province	Handgrip strength: electronic handgrip dynamometer Gait speed: assessed by measuring time to walk 15 feet at rapid pace on flat indoor surface	MCI assessed using MMSE MCI defined as MMSE scores lower than 17, 20, and 22 points for participants whose levels of education were illiterate, primary school, and middle school or above	671 participants developed MCI (21.6%) Generalized estimating equation adjusted for gender, BMI, age, and other confounders of the participants • Low handgrip strength: OR 1.78 (95% CI 1.51–2.10), *p* < 0.001 • Low gait speed: OR 1.71 (95% CI 1.45–2.01), *p* < 0.001 Association of handgrip strength combined with gait speed after adjusting for gender, BMI, age, and other confounders • Low handgrip strength + normal gait speed: OR 1.85 (95% CI 1.53–2.23), *p* < 0.001 • Normal handgrip strength + low gait speed: OR 1.74 (95% CI 1.43–2.12), *p* < 0.001 • Low handgrip strength + low gait speed: OR 2.40 (95% CI 1.83–3.15), *p* < 0.001

Table only includes results related to analyses on incident MCI, whenever possible; original studies may also include additional results, e.g. related to incident dementia

*AD* Alzheimer’s disease, *ADL* activities of daily living, *APOEε4* Apolipoprotein Ɛ4, *BMI* body mass index, *CES-D* Center for Epidemiologic Studies-Depression, *CI* confidence interval, *CIRS* Cumulative Illness Rating Scale, *CIRS-G* Cumulative Illness Rating Scale-Geriatric, *GDS* Geriatric Depression Scale, *HR* hazard ratio, *ICD* International Classification of Diseases, *KES* Knee extension strength, *LSMM* low skeletal muscle mass, *m/s* meters/second, *MCI* mild cognitive impairment, *MMSE* Mini Mental Status Examination, *MoCA* Montreal Cognitive Assessment, *MVPA* moderate to vigorous physical activity, *NIA-AA* National Institute on Aging-Alzheimer’s Association, *n.s.* not significant, *OR* odds ratio, *POMA* Performance Oriented Mobility Assessment, *PTSD* posttraumatic stress disorder, *rev.* reversed, *SD* standard deviation, *sec* seconds, *SPPB* Short Physical Performance Battery, *TUG* Timed Up and Go, *WHO* World Health Organization, *Wmax/kg* maximal aerobic workload in Watt per kilogram body weight

### Quality of included studies

Eight studies received the highest score of 9 [39, 41, 42, 44, 47, 49, 53, 54], five studies a score of 8 [40, 48, 51, 52, 55], four a score of 7 [37, 43, 46, 50], and two studies received a score of 6 [38, 45]. Overall, the quality of included studies can thus be rated as good, with only six out of 19 studies receiving a fair quality rating. Please refer to Table 2 for an overview of quality of included studies.

**Table 2:** Quality rating of included studies using Newcastle Ottawa scale

Study	Selection				Comparability	Outcome			Total Score (of 9)	Power
	Representativeness of exposed cohort (Max.: ☆)	Selection of non-exposed cohort (Max.: ☆)	Ascertainment of exposure (Max.: ☆)	Demonstration that outcome of interest was not present at study start (Max.: ☆)	Comparability of cohorts based on design or analysis (Max.: ☆☆)	Assessment of outcome (Max.: ☆)	Follow-up length sufficient for outcomes (Max.: ☆)	Adequacy of follow up of cohorts (Max.: ☆)		
Beeri et al. [41]	✫	✫	✫	✫	✫✫	✫	✫	✫	9	good
Beeri et al. [42]	✫	✫	✫	✫	✫✫	✫	✫	✫	9	good
Boyle et al. [52]	✫	✫	✫	✫	✫✫	✫	-	✫	8	good
Boyle et al. [49]	✫	✫	✫	✫	✫✫	✫	✫	✫	9	good
Byun et al. [40]	✫	✫	✫	✫	✫✫	✫	**-**	✫	8	good
Feng et al. [38]	✫	✫	✫	✫	✫✫	**-**	**-**	**-**	6	poor
Hooghiemstra et al. [37]	-	✫	✫	✫	✫✫	✫	**-**	✫	7	good
Lipnicki et al. [55]	✫	✫	✫	✫	✫✫	✫	✫	**-**	8	good
Luo et al. [43]	✫	✫	✫	✫	✫✫	**-**	✫	**-**	7	poor
Moon et al. [44]	✫	✫	✫	✫	✫✫	✫	✫	✫	9	good
Ng et al. [51]	✫	✫	✫	✫	✫✫	✫	**-**	✫	8	good
Nyberg et al. [39]	✫	✫	✫	✫	✫✫	✫	✫	✫	9	good
Pellecchia et al. [45]	-	✫	✫	✫	✫✫	✫	**-**	**-**	6	poor
Rosso et al. [53]	✫	✫	✫	✫	✫✫	✫	✫	✫	9	good
Salinas-Rodriguez et al. [46]	✫	✫	✫	-	✫✫	✫	✫	**-**	7	good
Sattler et al. [47]	✫	✫	✫	✫	✫✫	✫	✫	✫	9	good
Tian et al. [54]	✫	✫	✫	✫	✫✫	✫	✫	✫	9	good
Werneck et al. [48]	✫	✫	✫	✫	✫✫	**-**	✫	✫	8	good
Yang et al. [50]	✫	✫	✫	✫	✫✫	**-**	**-**	✫	7	good

### Associations between strength and variables related to muscle quality and function with incident MCI

The association between handgrip strength and incident MCI was examined in twelve studies: In six studies, higher handgrip strength was statistically significantly associated with lower risk of incident MCI [42, 43, 48] or lower handgrip strength with increased risk of MCI [45, 50, 52], whereas five studies did not report significant associations between handgrip strength and incident MCI [37, 41, 44, 46, 47]. One study reported a significant association between handgrip strength and incident MCI in women but not men [38]. Similarly, skeletal muscle mass was not associated with incident MCI in three studies [41, 44, 46], and neither was leg strength [51]. One study [42] reported that hand dexterity was associated with decreased risk of incident MCI, and this association remained significant when other motor domains (i.e., hand strength, gait function, and leg strength and balance) were included in the same model. Furthermore, one study created a muscular strength score derived from tests of 11 muscle groups [52] and showed that higher muscle strength was statistically significantly associated with a decreased risk of both incident and persistent MCI. Similarly, one study [41] provided evidence of an association between a higher composite sarcopenia score (albeit only including variables related to muscle mass and function) and increased MCI risk.

### Associations between endurance/cardiovascular fitness with incident MCI

Only one study [39], albeit with a large sample of nearly 1 million persons and a mean follow-up of 25.7 years examined the associations between cardiovascular fitness as assessed using a graded cycle ergometer test and incident MCI. The investigators reported that participants with low or medium as compared to high cardiovascular fitness at the age of 18 years had a statistically significantly increased risk of incident MCI, with point estimates ranging between 2.96 and 3.83 for low, and between 1.52 and 1.75 for medium fitness, and depending on the type of adjustment (i.e., different confounding variables were used in the analyses).

### Associations between balance, mobility and gait-related variables with incident MCI

Two studies examined the associations between one leg stand and incident MCI, with one study reporting no association [42], and one study providing evidence of an association between passing a 15 s one leg stand test and decreased risk of incident MCI [47]. One study [51] reported associations between worse performance in the Timed Up and Go test with increased risk of incident MCI, but associations between worse POMA score and incident MCI were only present in unadjusted models. Nine studies examined associations between different gait-related variables and the outcome of incident MCI. One study [42] reported that better gait function (as indicated by time and number of steps) was associated with a decreased risk of incident MCI. Similarly, six studies showed that lower gait speed was associated with increased MCI risk [49–51, 53, 55] or higher gait speed was associated with decreased MCI risk [43]. In one study [46], gait speed was not related to MCI risk, and in another [37], no overall associations between baseline gait speed and progression to MCI were found, but in analyses stratified by age, slower gait speed was associated with increased risk of new onset of MCI in participants aged > 65 years but not in those aged ≤ 65 years. One study [40] examined different gait variables as predictors of interest, and found that gait variability but not speed was associated with MCI risk. Specifically, participants with high gait variability had a 12% shorter mean survival free of MCI than participants with middle or low variability. Finally, one study [54] showed that each 0.05 m/s slower gait speed was associated with a 7% increase in the risk of developing MCI.

### Associations between global or composite physical fitness scores with incident MCI

One study [42] reported statistically significant associations between a higher global motor score (including hand dexterity, hand and leg strength, and gait function) with a decreased risk of incident MCI, i.e., each one standard deviation (SD) increase in global motor score at baseline was associated with about a 20% decreased risk of MCI. Further analyses revealed, however, that global motor function was only significantly associated with incident non-amnestic but not amnestic MCI. Another study [49] created a physical frailty composite score based on four components (i.e., grip strength, gait, body composition, and fatigue) and showed that a higher physical frailty score was statistically significantly associated with an increased risk of incident as well as persistent MCI. Similarly, one study [46] used a sarcopenia score including variables on skeletal muscle mass, gait speed, and handgrip strength and showed statistically significant associations between a higher sarcopenia score and an increased MCI risk. Finally, two studies [44, 45] reported that lower extremity physical functional impairment as assessed by the SPPB was associated with an increased risk of new onset of MCI.

**Fig. 2:**
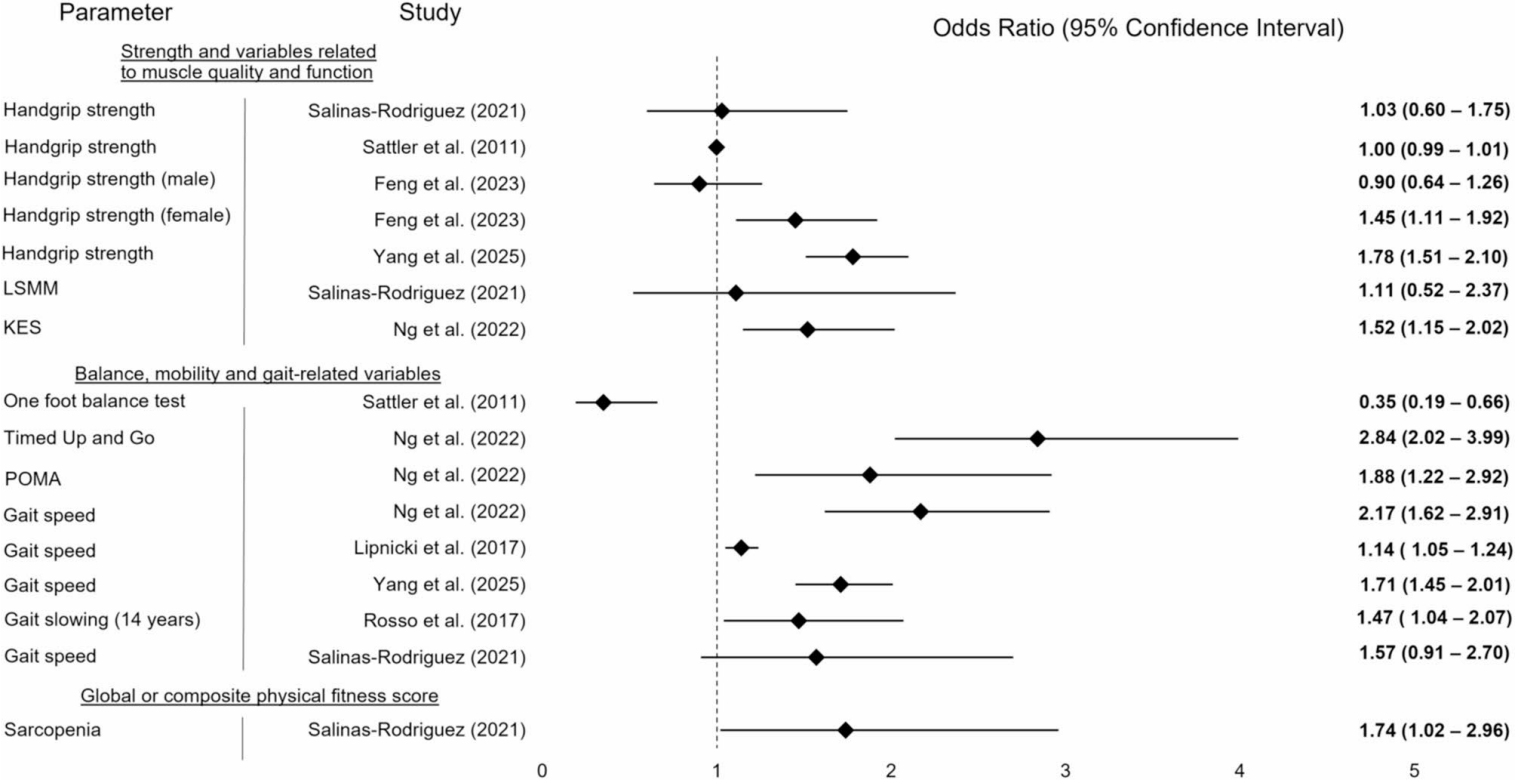
Graphical display of selected results from included studies reporting odds ratios. Note: Some studies reported multiple results for a motor performance parameter depending on the degree of adjustment (please also refer to Table 1); abbreviations: LSMM = Lean skeletal muscle mass; KES = Knee extension strength; POMA = Performance Oriented Mobility Assessment

**Fig. 3:**
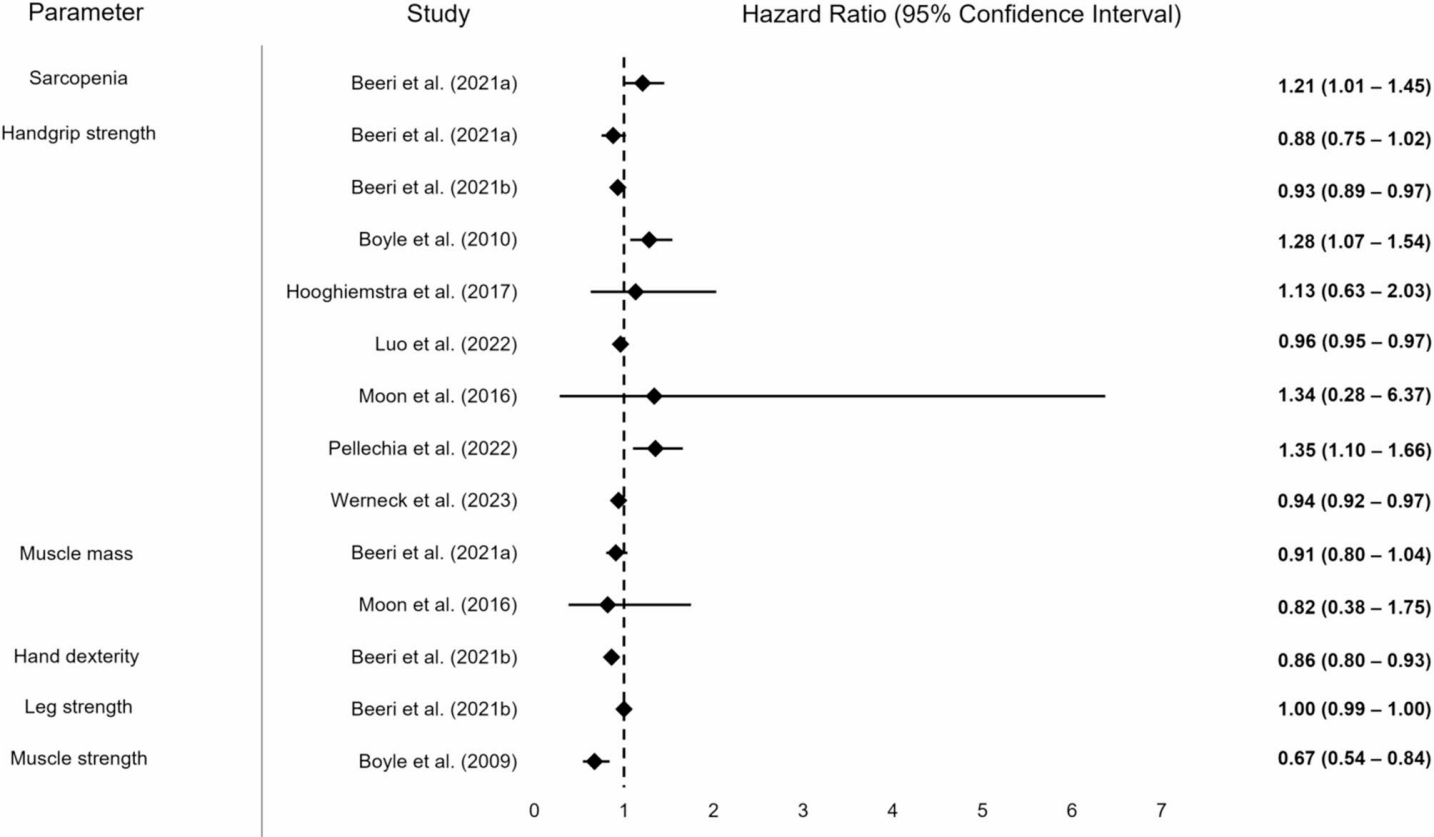
Graphical display of selected results from included study on strength, muscle quality and function reporting Hazard ratios. Note: Some studies reported multiple results for a motor performance parameter depending on the degree of adjustment (please also refer to Table 1)

**Fig. 4:**
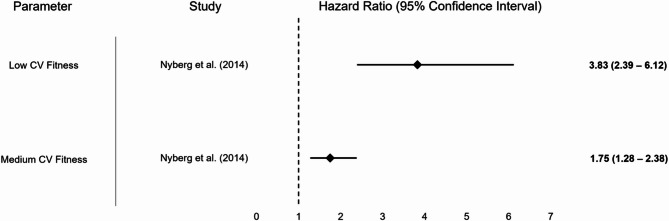
Graphical display of selected results from included study on endurance/ cardiovascular fitness reporting hazard ratios. Note: Some studies reported multiple results for a motor performance parameter depending on the degree of adjustment (please also refer to Table 1); abbreviations: CV = cardiovascular fitness

**Fig. 5:**
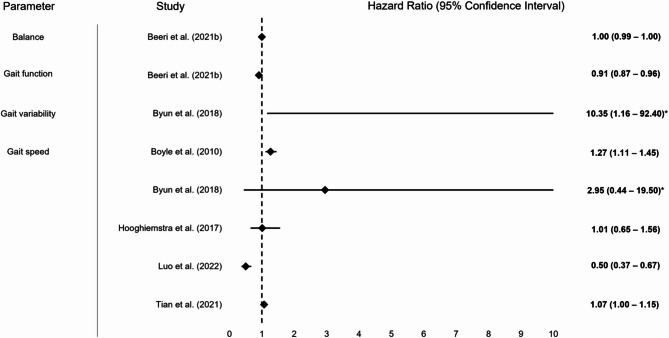
Graphical display of selected results from included study on balance, mobility and gait reporting Hazard ratios. Note: Some studies reported multiple results for a motor performance parameter depending on the degree of adjustment (please also refer to Table 1); Hazard ratios and 95% confidence intervals marked with an * are not fully depicted graphically due to higher upper bound of confidence interval

**Fig. 6:**
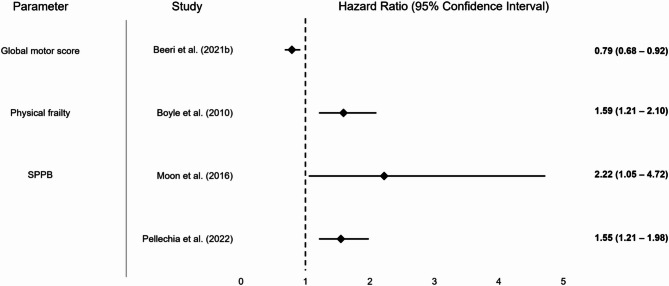
Graphical display of selected results from included study on global or composite fitness reporting hazard ratios. Note: Some studies reported multiple results for a motor performance parameter depending on the degree of adjustment (please also refer to Table 1); abbreviations: SPPB = Short Physical Performance Battery

For a graphical display of results of included studies, please refer to Figs. 2, 3, 4, 5 and 6.

## Discussion

We here provide an overview of the current state of research on different components of physical fitness or motor performance and the outcome of incident MCI. Higher cardiovascular and overall physical fitness was associated with a decreased risk of incident MCI. However, the associations between strength, balance- or gait-related variables and the risk of MCI are less clear, and there are also differences across studies related to the strength of observed associations and effect sizes.

Results on the associations between strength and incident MCI were inconsistent, e.g., six studies reported associations between lower handgrip strength with higher risk of incident MCI [45, 50, 52] or between higher handgrip strength and decreased risk of incident MCI [42, 43, 48], respectively; whereas five studies failed at establishing associations between handgrip strength and risk of new onset of MCI [37, 41, 44, 46, 47]. One study found an association between lower handgrip strength and increased risk of incident MCI in women but not men [38]. Skeletal muscle mass does not appear to be associated with MCI risk [41, 44, 46], whereas hand dexterity [42] and overall strength scores [52] were predictors of MCI risk. While cardiovascular fitness has been favorably associated with a variety of health outcomes in many research studies, we only found one longitudinal study that examined associations between cardiovascular fitness and risk of incident MCI [39]. As one may expect, the study provided evidence that lower cardiovascular fitness is associated with higher risk of incident MCI. With regard to balance and gait-related variables as potential predictors of incident MCI, results of included studies are inconsistent. Most studies focused on gait speed, and some found associations between slower gait speed and increased MCI risk [49–51, 53, 55], whereas others did not [46] or only in subgroups [37], i.e., persons aged > 65 years. In addition, gait function and variability were associated with the risk of incident MCI [40]. Finally, we also included studies that created global or composite physical fitness scores, and these studies all showed that higher overall fitness was associated with a decreased risk of incident MCI [42, 46, 49].

The inconsistent results regarding strength, balance, and gait-related variables and their association with risk of incident MCI can likely be attributed to several factors. First, differences in study design, such as variations in sample size and age, population characteristics including ethnic and/ or geographical background, and follow-up duration, may impact findings. Another factor that might explain inconsistent findings is that physical fitness parameters are very complex and may not directly correlate with cognitive decline in a linear fashion. Also, both physical fitness (predictor) and MCI (outcome) are known to be impacted by various mediating and confounding variables, such as medical comorbidities, lifestyle factors, or genetic predispositions, that are not accounted for in many studies. Differences in point estimates and effect sizes across studies could result from diverse analytical approaches, including but not limited to statistical methods, model adjustments, or the inclusion/ exclusion of confounders.

In addition to the 19 studies included in this review, there were some other longitudinal studies that are closely related to our research question but did not fulfill all pre-defined inclusion criteria. For example, investigators from Oregon reported that, among 204 cognitively unimpaired older adults, age-related changes in gait speed and finger-tapping speed differed significantly between participants who later developed MCI versus those who remained cognitively unimpaired [56]. An analysis based on data from 1478 cognitively unimpaired older adults participating in the Mayo Clinic Study of Aging showed that faster gait speed at baseline was associated with less pronounced decline in global and domain-specific cognitive scores after a mean follow-up of 4 years [57]. With regard to strength, Korean researchers found that a decline in handgrip strength over time was associated with higher odds of having MCI among over 6000 older adults [58]. Investigators who conducted a case-control retrospective study in the setting of the Baltimore Longitudinal Study of Aging provided evidence that a greater rate of increase in 400-m walk lap time variability differentiated individuals who progressed to MCI or Alzheimer’s disease from matched controls who remained cognitively unimpaired over a mean follow-up of 5 years [59]. Moreover, studies exist that examined the relationship between physical fitness and incident MCI in specific patients groups, e.g. in hemodialysis patients [60]. Furthermore, it is important to note that there are also longitudinal studies that did not find associations between physical fitness and cognitive impairment or decline [61].

In addition, several cross-sectional studies showed that various components of physical fitness or motor performance such as gait speed [62–66] and other gait-related parameters [67–70], grip strength [63, 71–73], sarcopenia [63, 74, 75] and frailty [76, 77], falls and fall risk [78], cardiorespiratory fitness and endurance [66, 79], balance and mobility [65, 80], as well as overall physical performance [81, 82] are associated with MCI risk or cognitive impairment, albeit some studies also reported no associations [83].

Our review is also in line with a prior review on the associations between physical fitness and the risk of incident dementia [33] which showed that decreased lower limb motor function but not handgrip strength was associated with increased risk of developing dementia. Since publication of this review, some additional studies have become available that also report associations between fitness and new onset of dementia, for example related to gait [84, 85]. Furthermore, in line with our observations, one systematic review reported associations between gait variability assessed using instrumented kinematic assessment and incident MCI [86].

Of note, relevant to our review, motoric cognitive risk is a predementia syndrome characterized by slow gait and cognitive complaints [87], that is also known to show overlap with MCI as well as frailty [88]. Persons with motoric cognitive risk have an increased risk of developing incident dementia [89–91].

In addition, in our review, we did not include studies on the associations between body composition or related variables (other than muscle mass which is an indicator of muscle strength) and the risk of incident MCI. While body composition is considered a part or component of physical fitness in some definitions such as from the ACSM [1], we referred to other definitions that do not include body composition as a central component of physical fitness [2, 3] when we created the search terms for this review. However, in a non-systematic literature search, we identiefied several studies on the associations between different variables related to body composition such as body mass index [92–95] or central obesity [96] and the risk of incident MCI. In general, those studies showed that a less favorable body composition (i.e., higher body mass index or higher central obesity) is associated with increased risk of incident MCI, albeit one study found inverse results [97].

This review may have implications for clinical practice. For example, physicians treating older adults at risk for cognitive impairment may want to emphasize on the importance of engaging in physical fitness-enhancing activities and may even include a brief physical fitness exam to detect limitations of their patients with regard to certain fitness components such as cardiovascular fitness or balance. Such fitness components could be improved by engaging in targeted, individualized physical exercise. Future research should focus on examining the associations between different components of physical fitness with risk of incident MCI. Most studies included in our review focused on strength and gait-related parameters as potential predictors of MCI, with most studies using handgrip strength or gait speed, and less attention has been paid to other fitness components such as cardiovascular fitness, balance, mobility, or gross-motor coordination. Thus, any conclusions on the associations between these fitness components and the risk of incident MCI may be premature, and should be considered preliminary until confirmed by future studies.

It has been proposed in the literature that physical fitness, particularly cardiorespiratory fitness, can lead to better cognition and decreased MCI risk through various mechanisms, including but not limited to an increased neural plasticity [98] and cerebral oxygenation, or improved endothelial function. To this end, a meta-analysis including 51 randomized controlled trials revealed that not only endurance training, but also resistance training, may lead to improved endothelial function [99]. Resistance training has also been linked to reduced serum homocysteine [100], and increased insulin-like growth factor 1 (IGF-1) [101, 102]. While an elevated level of homocysteine is associated with impaired cognition [103] and Alzheimer’s disease [104], IGF-1 is known to have a favorable effect on neuronal growth and cognitive performance [105]. Physical activity may also play an important role in the association between physical fitness and incident MCI. For example, one study included in our review [54] showed that physical activity fragmentation (described by the investigators as the degree to which an individual alternates physical activity bouts and periods of rest) may interact with gait in predicting MCI risk, i.e., each 0.05 m/s slower gait speed at low but not high activity fragmentation was associated with MCI risk. The researchers postulate that slow gait speed coupled with low degree of activity fragmentation may be indicative of underlying causes linked to the central nervous system such as poor balance, gross-motor coordination or motor planning, which may ultimately be related to impaired cognition [54].

To the best of our knowledge, our review is novel in that it examined the associations between various components of physical fitness or motor performance with the risk of incident MCI in older adults based on longitudinal, observational studies. However, as in any review, one limitation pertains to the search term which may not have been suitable to detect all pertinent publications. However, we created the search term by considering search terms used in previous literature reviews on similar topics. Furthermore, the initial search was conducted in only one scientific database, i.e., PubMed and may therefore have missed pertinent studies that are not listed in PubMed, albeit we also screened reference lists of included research. However, when we updated the literature search, we also searched in Scopus and Web of Science databases. In our review, we included studies that examined whether physical fitness or motor performance (as predictor variables) is associated with the risk of incident MCI (as outcome of interest), hypothesizing that physical fitness may have an impact on future MCI risk. However, reverse causality is also possible, and cannot be ruled out based on observational studies like those included in our review. This means that it is also possible that persons who develop MCI are more likely to have lower fitness levels in pre-MCI stages, potentially due to lower engagement in physical activity and exercise. Indeed, longitudinal studies have shown that having MCI is associated with greater decline in physical fitness as compared to being cognitively unimpaired [106]. In addition, while we focused on different components of physical fitness or motor performance as predictor variables, we did not consider all variables that may be considered to be part of physical fitness. For example, we did not include studies on the associations between body composition and the risk of MCI in our review, e.g., [92–94]. Finally, we used the Newcastle-Ottawa Scale to rate the quality of included studies in terms of reporting, but did not evaluate risk of bias.

## Conclusions

Higher cardiovascular and overall physical fitness appears to be associated with a lower risk of incident MCI in older adults, whereas associations between strength and balance- or gait-related variables with MCI risk are inconsistent. Importantly, no study reported that higher fitness is associated with increased MCI risk. Therefore, in line with a large body of prior research, our review suggests that older adults should aim at maintaining or increasing their physical fitness or motor performance in order to potentially delay new onset of MCI. More research is needed to confirm these observations, particularly focusing on cardiovascular fitness, balance, mobility, or gross-motor coordination as predictor variables, and also explore underlying mechanisms.

## Supplementary Information

Supplementary Material 1.

Supplementary Material 2.

## Data Availability

All manuscripts included in the systematic review are published and available from PubMed.
