# Supplementary material for: Physical fitness and incident mild cognitive impairment: a systematic review
Source: Eur Rev Aging Phys Act. 2025 Jun 14;22:10. doi: 10.1186/s11556-025-00376-9 (PMC12166603; doi:10.1186/s11556-025-00376-9)
Supplement: Supplementary file 1 — Supplementary Material 1. [file 11556_2025_376_MOESM1_ESM.docx]

**Supplementary Material 1: Search terms by database**

**PubMed**:

*("motor performance*"[Title/Abstract] OR "motor skill*"[Title/Abstract] OR "motor test*"[Title/Abstract] OR "motor abilit*"[Title/Abstract] OR "fitness"[Title/Abstract] OR "strength"[Title/Abstract] OR "endurance"[Title/Abstract] OR "balance"[Title/Abstract] OR "gait"[Title/Abstract] OR "walk*"[Title/Abstract] OR "coordinati*"[Title/Abstract] OR "speed"[Title/Abstract] OR "flexibilit*"[Title/Abstract] OR "range of motion"[Title/Abstract]) AND ("mild cognitive impairment"[Title/Abstract] OR "MCI"[Title/Abstract])*

**Scopus:**

*TITLE-ABS ( motor AND performance* ) OR TITLE-ABS ( motor AND skill* ) OR TITLE-ABS ( motor AND test* ) OR TITLE-ABS ( motor AND abilit* ) OR TITLE-ABS ( fitness ) OR TITLE-ABS ( strength ) OR TITLE-ABS ( endurance ) OR TITLE-ABS ( balance ) OR TITLE-ABS ( gait ) OR TITLE-ABS ( walk ) OR TITLE-ABS ( coordinat* ) OR TITLE-ABS ( speed ) OR TITLE-ABS ( flexibilit* ) OR TITLE-ABS ( range AND of AND motion ) AND TITLE-ABS ( mild AND cognitive AND impairment ) OR TITLE-ABS ( mci )*

**Web of Science:**

*((((((((((((((TI=(motor performance*)) OR Ti=(motor skill*)) OR TI=(motor test*)) OR TI=(fitness)) OR TI=(strength)) OR TI=(motor abilit*)) OR TI=(endurance)) OR TI=(balance)) OR TI=(gait)) OR TI=(coordinati*)) OR TI=(speed)) OR TI=(flexibilit*)) AND TI=(mild cognitive impairment)) OR TI=(MCI))*
