# Supplementary material for: Physical fitness and incident mild cognitive impairment: a systematic review
Source: Eur Rev Aging Phys Act. 2025 Jun 14;22:10. doi: 10.1186/s11556-025-00376-9 (PMC12166603; doi:10.1186/s11556-025-00376-9)
Supplement: Supplementary file 2 — Supplementary Material 2. [file 11556_2025_376_MOESM2_ESM.docx]

**Supplementary Material 2: Cross table providing an overview of variables examined in included studies**

| **Variable**  **Study** | Strength and variables related to muscle quality and function | Endurance / cardiovascular fitness | Balance, mobility and gait-related variables | Global or composite fitness scores |
| --- | --- | --- | --- | --- |
| Beeri et al. (40) | X |  |  |  |
| Beeri et al. (41) | X |  | X | X |
| Boyle et al. (51) | X |  |  |  |
| Boyle et al. (48) |  |  | X | X |
| Byun et al. (39) |  |  | X |  |
| Feng et al. (37) | X |  |  |  |
| Hooghiemstra et al. (36) | X |  | X |  |
| Lipnicki et al. (54) |  |  | X |  |
| Luo et al. (42) | X |  | X |  |
| Moon et al. (43) | X |  |  | X |
| Ng et al. (50) | X |  | X |  |
| Nyberg et al. (38) |  | X |  |  |
| Pellecchia et al. (44) | X |  |  | X |
| Rosso et al. (52) |  |  | X |  |
| Salinas-Rodriguez et al. (45) | X |  | X | X |
| Sattler et al. (46) | X |  | X |  |
| Tian et al. (53) |  |  | X |  |
| Werneck et al. (47) | X |  |  |  |
| Yang et al. (49) | X |  | X |  |
